# *In Vitro* Potential of the Acetone Leaf Extract and Fractions of *Psychotria capensis* (Eckl.) Vatke (Rubiaceae) to Combat Co-Infection of Tuberculosis and Helminthiasis

**DOI:** 10.3389/fphar.2021.744137

**Published:** 2022-01-11

**Authors:** Abimbola O. Aro, Ibukun M. Famuyide, Ademola A. Oyagbemi, Prudence N. Kabongo-Kayoka, Lyndy J. McGaw

**Affiliations:** ^1^ Department of Agriculture and Animal Health, College of Agriculture and Environmental Science, University of South Africa, Florida, South Africa; ^2^ Phytomedicine Programme, Department of Paraclinical Sciences, Faculty of Veterinary Science, University of Pretoria, Pretoria, South Africa; ^3^ Department of Veterinary Physiology and Biochemistry, Faculty of Veterinary Medicine, University of Ibadan, Ibadan, Nigeria

**Keywords:** *Psychotria capensis*, Rubiaceae, antimycobacterial, anthelmintic, immunomodulatory, tuberculosis, helminth, co-infection

## Abstract

Tuberculosis (TB) is a disease of global importance that affects millions of people. Approximately a quarter of the world’s population is currently infected with *M. tuberculosis*, and about 10% of those infected will develop into active disease, particularly immune compromised individuals. Helminthiasis is of global health importance, affecting over 2 billion people mostly in resource-poor countries. Co-infection with tuberculosis (TB) and helminths (worms) is an emerging global public health concern with both affecting about one-third of the global population. Chronic infection with helminths can result in impaired immune responses to TB as well as enhancing failure to TB therapy and BCG vaccination. Antimycobacterial and anthelmintic activities of the acetone extract and fractions of *Psychotria capensis* were evaluated, including their *in vitro* safety. In addition, the anti-inflammatory and immunomodulatory effect of the fractions and crude extract of *P. capensis* were assessed. Antimycobacterial activity of the extract and fractions was tested against four non-tuberculous mycobacteria (*Mycobacterium smegmatis*, *M. fortuitum*, *M. aurum, M. bovis* BCG) and pathogenic *M. tuberculosis* H37Rv while the Egg Hatch Assay (EHA) was used for the anthelmintic test on eggs of *Haemonchus contortus*. Cytotoxicity was determined against Vero kidney cells while *in vitro* immune modulation via cytokine production was determined on activated macrophages. The minimum inhibitory concentration (MIC) values of the *Psychotria capensis* acetone extract and fractions ranged from 39 to 1,250 μg/ml with the crude extract and hexane fraction having the best MIC values (both 39 μg/ml). In the EHA, the inhibitory concentration (IC_50_) ranged from 160 to 630 μg/ml with the hexane fraction having the best activity. The hexane and chloroform fractions were relatively non-toxic with LC_50_ values of 290 and 248 μg/ml respectively, while the acetone crude extract (64 μg/ml) and n-butanol fraction (71 μg/ml) were moderately toxic. The SI values (LC_50_/MIC) ranged from 0.1 to 7.4 with the hexane fraction having the highest value against *M. smegmatis* (7.4). The hexane fraction had the best dual anthelmintic and antimycobacterial activity. This fraction had the best NO inhibitory activity and was the least cytotoxic, indicating that its activity was not due to general metabolic toxicity, with 96.54% cell viability. Pro-inflammatory cytokines such as IL-12p70 were upregulated while IL-10 expression was inhibited by the extracts. Compounds were detected using GC-MS analysis, and in both the crude acetone extract and the hexane fraction was the diterpene neophytadiene, which has anti-inflammatory and antimicrobial activity. Finding alternative or complementary approaches to dealing with TB infections by, amongst other things, reducing the incidence of helminth infestations may lessen the burden of TB, contributing to slowing the spread of multi-drug resistance.

## Introduction

Tuberculosis (TB) is still a major cause of death worldwide with the majority of the cases occurring in Asia and Africa (Zaman, 2010). Helminths are parasitic worms and are one of the most common infectious agents in developing countries (Hotez et al., 2008). Helminthiasis is of global health importance, affecting over 2 billion people mostly in resource-poor countries (Allen and Maizels, 2011). There is considerable geographical coincidence of these two diseases with the majority of the infections reported in resource-poor countries of the world (Salgame et al., 2013). The ferocity of TB in developing and underdeveloped countries where helminth infections are prevalent may be due to the co-infection with helminths, with such parasitic infestations enhancing the pathogenesis of mycobacterial infections (Elias et al., 2001; Abate et al., 2012). Co-infection with helminths can result in impaired immune responses to TB as well as enhanced failure to TB therapy and BCG vaccination (Borkow and Bentwich, 2004; Resende Co et al., 2007). Therefore, communities where helminth infection is endemic could also have a high morbidity and mortality rate of TB, contributing towards promoting the development of drug resistance.

More than 1.5 billion people, or 24% of the world’s population, are infected with soil-transmitted helminths worldwide (WHO, 2020). Helminth infections in humans can be chronic but seldom lethal and can last for decades. However, they can cause various symptoms ranging from abdominal pain, diarrhoea, malaise and anaemia to malnutrition and impaired physical and cognitive development, particularly in children (King, 2010; Riaz et al., 2020; WHO, 2020). Parasitic infections in production animals cause significant direct and indirect economic losses. For example, haemonchosis, a significant and common parasitic infection of small ruminants caused by the nematode *Haemonchus contortus*, leads to anaemia, primarily due to the blood-sucking activity, and consequently a significant reduction in the production of infected animals which includes a decrease in the growth of young animals and occasional death of the infected animals (Arsenopoulos et al., 2021).

The modulation of T-cell mediated immune responses to TB can be induced by helminth co-infection (Metenou et al., 2012). Moreover, larvae of many intestinal helminth parasites have the ability to migrate through the lungs, thereby inducing the host immune response to support the pathogenesis of TB (George et al., 2015). Studies have shown that in an anti-inflammatory environment, worm co-infection weakens both protective and immunopathological responses to *M. tuberculosis,* thereby promoting the widespread distribution and severity of TB especially in resource-poor countries (Tristão-Sá et al., 2002; Abate et al., 2012). It was further demonstrated that a profound T helper 2 (Th-2) immune response induced by intestinal helminths in the host can inhibit the production of the T helper 1 (Th-1) immune response that is imperative to combat mycobacterial infections (Diniz et al., 2010; Abate et al., 2012). Therefore, there is an urgent need to identify and develop drug candidates with potential to inhibit the growth of both parasitic helminths and pathogenic mycobacteria, especially in countries where helminth infections and TB are endemic. This will potentially assist with reducing the growing threat of drug resistant TB in terms of reducing the incidence of infections and need for treatment.

From antiquity, different plant parts have been used traditionally to treat various infectious diseases. This knowledge can be harnessed for drug development to identify potential drug leads or novel templates. Despite the decline in the amount of funding available for natural products-based drug discovery programs, studies conducted by different research groups across the globe have revealed the pertinent roles that natural products can play in drug discovery and development (Newman and Cragg, 2020). Therefore, harnessing the unexploited scaffolds of natural products could be a useful tool in advancing drug discovery (Ortholand and Ganesan, 2004). Globally, crude plant extracts are used to treat many infections and diseases owing to the abundance of metabolites such as coumarins, alkaloids, flavonoids, chalcones, phenols, lignans, simple aromatics and peptides present in them (Copp and Pearce, 2007; García et al., 2012). The use of medicinal plants in the treatment of helminth parasites is a common practice, especially in developing countries including South Africa (Waller et al., 2001; Aremu et al., 2012; Adamu et al., 2013). Also, medicinal plants are used in many parts of southern Africa to treat TB-related symptoms including chest complaints, respiratory ailments, fever and coughing (McGaw et al., 2008).

Various plant species from the Rubiaceae family are promising sources of new bioactive compounds which may lead to the development of new products as active molecules or drug prototypes due to their structural diversity and pharmacological activities (Martins and Nunez, 2015). One peculiar characteristic of this family is that they contain a wide range of secondary metabolites, such as alkaloids, flavonoids, terpenes, anthraquinones and coumarins, with good pharmacological properties (Heitzman et al., 2005). These classes of secondary metabolites have been associated with antimicrobial, antiparasitic, anti-malarial, hepatoprotective, antioxidant, and other biological activities (Martins et al., 2013). Decoctions of the roots of *Psychotria capensis* (Eckl.) Vatke are used as emetics and to treat gastric complaints in southern Africa (Watt et al., 1962; Pooley, 1993). However, no literature reporting the anthelmintic efficacy, or other activities potentially related to gastric disorders, of *P. capensis* is available. The acetone crude extract of leaves of *P. capensis* has been previously reported to have excellent antimycobacterial, antioxidant and anti-inflammatory activities (Aro et al., 2016), motivating further investigation of this species. In this study, the antimycobacterial and anthelmintic activities, immune modulatory effects, and cytotoxicity of acetone crude leaf extracts of *P. capensis* and its fractions obtained by solvent-solvent fractionation were determined.

## Materials and Methods

### Plant Collection and Identification

The leaves of *Psychotria capensis* were collected from the University of Pretoria, Hatfield campus. A voucher specimen was prepared and deposited after being identified at the H.G.W.J. Schweickerdt Herbarium, University of Pretoria, South Africa. The voucher number is PRU 120875. Leaves were air-dried at room temperature, ground to fine powder in a Macsalab mill (model 2000 LAB Eriez) using a sieve of 1 mm diameter, and stored in closed glass containers in the dark until needed.

### Crude Extraction and Solvent-Solvent Fractionation

Five hundred grams of the ground leaves of *P. capensis* were extracted with 5 L of acetone. The mixture was mixed thoroughly and left overnight at room temperature. The supernatant was then filtered and concentrated to dryness. This was repeated thrice for maximum extraction. Acetone was chosen as the extracting solvent because of its ability to extract compounds of a wide range of polarity (Eloff, 1998b; Famuyide et al., 2019). Eloff (1998b) stated that acetone extracted both polar and non-polar inhibitors of bacterial growth, as could be seen from biochromatograms using different solvent systems of varying polarity. Fractionation of the crude extract was done through sequential partitioning as previously described (Eloff, 1998b) with a slight modification. The dried acetone crude extract was dissolved in 300 ml of chloroform and sonicated to enhance dissolution. The solution was poured into a separating funnel (2.5 L) and 300 ml of distilled water was added. The mixture was shaken gently and allowed to separate into aqueous and chloroform fractions for 24 h. The chloroform fraction was removed, leaving the aqueous fraction. The aqueous fraction was successively partitioned with hexane (300 ml) and butanol (300 ml). This afforded three solvent fractions after concentration using a rotary evaporator. These were hexane, butanol and aqueous fractions. The chloroform fraction initially collected in the first step was transferred back into the separating funnel and 300 ml of 35% water in methanol was added, mixed gently, and allowed to settle for 24 h. Two layers were formed with the 35% water in methanol fraction at the top and the chloroform fraction at the bottom. Both fractions were removed and concentrated.

### GC-MS Analysis of the Crude Extract and Hexane Fraction of *Psychotria capensis*

An Agilent 7890B GC system gas chromatograph equipped with a split/splitless injector and a 5977B GC/MSD single-quadrupole GC/MS instrument mass spectrometer (MS) with a NIST 14 library were used. An injection port equipped with a 1 mm internal diameter liner operated in splitless mode (after 1 min, split ratio was 1:20) was kept at a temperature of 250°C and a pressure of 63 kPa. An Agilent 122-7032UI: DB-WAX UI, 20–250°C (max 260°C) (30 m × 250 µm × mm × 0.25 µm), column was used for separation. The oven temperature was set at 40°C at a hold-up time of 4 min, then a ramp of 8 C/min to 220°C followed by a hold time of 5 min, respectively. Helium at a constant flowrate of 1.2 ml/min, a velocity of 39.723 cm/s at a pressure of 63.057 kPa, with a hold-up time of 1.2587 was the carrier gas used. The injection port and transfer line temperatures were set at 250°C and electron impact ion source was held at 230°C. The mass spectrometer (MS) was operated in a full scan mode (m/z 40–400) and in a selected ion monitoring (SIM) mode. A solvent delay time of 4 min was used. Ions for detection of individual analytes in SIM mode were selected using the mass spectra of standards generated in SCAN mode at a scan speed of 1.562 (N = 2) and frequency of 2.6.

### Antimycobacterial Assay

A microplate two-fold serial dilution method was used to determine the minimum inhibitory concentration (MIC) values of the crude plant extract and fractions (Eloff, 1998a; McGaw et al., 2008). The acetone extract and fractions from *P. capensis* were screened against *Mycobacterium smegmatis* (ATCC 1441), *M. fortuitum* (ATCC 6841), *M. aurum* (NCTC 10437), *M. bovis* BCG (Pasteur strain P1172) and virulent laboratory strain *M. tuberculosis* H37Rv (ATCC 27294). A stock solution of 10 mg/ml of the crude and fractions were dissolved in acetone to prepare a working solution as acetone is relatively non-toxic to the test organisms (Eloff, 1998b). Briefly, 100 µl of the solutions were serially diluted with OADC-supplemented Middlebrook 7H9 broth in 96-well microtitre plates before mycobacterial culture (100 µl) was added to each well. Rifampicin (RIF) was included as positive control; acetone was used as a solvent control while broth only served as sterility control. Concentrations (0.01–2.5 mg/ml) were tested at least in triplicate, and the entire experiment was repeated. The fast-growing *M. smegmatis* and *M. fortuitum* were incubated at 37°C overnight and 48 h respectively, while *M. aurum*, *M. bovis* BCG and H37Rv cultures were incubated for 5–7 days. Minimum inhibitory concentration (MIC) values were determined using a tetrazolium violet (INT) indicator. The colour reaction after addition of INT generally occurred after 30 min to 1 h incubation. The lowest concentration of crude extract and fractions that inhibited the growth of the mycobacteria, resulting in a visible decrease in production of the red formazan was recorded as the MIC value. The total antibacterial activity (ml/g) of the extracts was calculated as the total mass (mg) of the extract divided by the MIC value (mg/ml) (Eloff, 2004). The TAA was not relevant for both controls, positive (antibiotic) and negative (acetone).

### Anthelmintic Assay

#### Recovery and Preparation of *Haemonchus contortus* Eggs

The studies involving animals were reviewed and approved by the Research Ethics Committee, University of Pretoria, approval number REC022-18. The eggs were prepared according to the protocol of the World Association for the Advancement of Veterinary Parasitology (WAAVP) (Coles et al., 1992) with some modifications (Adamu et al., 2013). A clinically healthy, worm-free sheep housed on concrete floor indoor, fed hay and commercial concentrate pellets with free access to water was experimentally infected with mono-specific larval suspensions of fresh *Haemonchus contortus* (obtained from MSD Animal Health, South Africa)*.* Eggs were recovered from faeces of the sheep by making it into a slurry using a mortar and pestle. The slurry was then filtered through sieves of pore sizes 250 μm, 150 μm, 63 μm and 38 µm respectively. The suspension retained on the 38 µm sieve was washed into 50 ml clean centrifuge tubes and resuspended in a magnesium sulphate solution prepared at a specific gravity of 1.10 and centrifuged at 1,000 × g for 10 min to further separate eggs from faecal debris. The resultant supernatant was then filtered through the 38 µm sieve to collect the eggs which were gently washed with distilled water to remove the salts. The eggs were viewed under a microscope and brought to a final concentration of 100 eggs per 0.2 ml.

#### Egg Hatch Assay

The Egg Hatch Assay (EHA) was used to determine anthelmintic activity on eggs of *Haemonchus contortus* (Coles et al., 1992). Briefly, egg suspension (0.2 ml) was distributed in wells of a 48-well flat-bottomed microplate, so that each well contained approximately 100 fresh eggs, and mixed with the same volume of plant extract dissolved in acetone at an initial concentration of 10 mg/ml in six serial dilutions (2,500, 1,250, 625, 312.5, 156, and 78 μg/ml). The positive control, albendazole was dissolved in 1% DMSO (concentration range of 0.078–0.005 μg/ml). The negative control plates contained 200 µl PBS, and 200 µl egg suspension. The eggs were incubated in this mixture for 48 h at 25°C and 70% relative humidity. Post incubation, a drop of Lugol’s iodine solution (Riedel de Haen, Germany) was added to stop further hatching of the eggs. All the eggs and first-stage larvae (L1) in each plate were counted. Three replicates for each concentration and control were used. The percent inhibition of egg hatching was calculated using the formula below (Coles et al., 1992; Bizimenyera et al., 2006):

$$\text{Inhibition \% }=\;100\;\left({1-\text{P}}_{\text{test}}{-\text{P}}_{\text{control}}\right)$$

where P_test_ is the number of eggs hatched or larval forms (L1) after incubation with test substance, and P_control_ is the respective numbers in the negative control. The concentration that inhibited egg hatching by 50% (IC_50_) was calculated from the percentage inhibition values against the concentration. Mean values were calculated.

### Cytotoxicity Assay

The cytotoxicity of the crude plant extract and fractions was tested against Vero African green monkey kidney cells purchased from the American Type Culture Collection (ATCC^®^ CCL-81™). The 3-(4,5-dimethylthiazol)-2,5-diphenyl tetrazolium bromide (MTT) assay (Mosmann, 1983) was used with slight modifications (McGaw et al., 2007). Cells were maintained in minimal essential medium (MEM, Highveld Biological, South Africa) supplemented with 5% foetal calf serum (Adcock-Ingram) and 0.1% gentamicin (Virbac) in a 5% CO_2_ incubator. Cell suspensions from 70 to 80% confluent monolayer cultures were plated at a density of 5 × 10^4^ cells into sterile flat-bottomed 96-well microtitre cell culture plates and incubated for 24 hat 37°C in a 5% CO_2_ incubator before exposure to the extracts. The extract and fractions were dissolved in acetone at a concentration of 100 mg/ml, and appropriate dilutions were prepared in MEM and added to the wells. Cells were exposed to various concentrations (0.025–1 mg/ml) of plant extracts for 48 h. Doxorubicin (Pfizer) and acetone were included as positive and negative controls, respectively. After the incubation period, wells were rinsed with phosphate buffered saline (PBS, Sigma) after which fresh medium was added into the wells. Then 30 µl (5 mg/ml) of MTT dissolved in PBS was added to each well and the plates were further incubated for 4 h at 37°C. After this, the medium from the wells was discarded and 50 µl of DMSO was added to the wells to dissolve the formed formazan crystals. Absorbance was measured on a microplate reader (BioTek Synergy) at a wavelength of 570 nm. Each extract concentration was tested in quadruplicate and the assay was repeated twice. The concentration causing 50% inhibition of cell viability (LC_50_) was calculated. Selectivity index (SI) values were obtained by dividing LC_50_ values by the MIC values and EC_50_ values for antimycobacterial and anthelmintic activities respectively.

### Nitric Oxide Inhibition Assay

#### Preparation of Cells

RAW 264.7 macrophage cells (ATCC, CRL-2278) were cultured in cell culture flasks using Dulbecco’s Modified Eagle’s Medium (DMEM; Sigma) containing L-glutamine supplemented with 10% foetal bovine serum (FBS, Gibco) and 1% penicillin/streptomycin/fungizone (PSF; Sigma) at 37°C with 5% CO_2_. Cells were seeded at a concentration of 0.2 × 10^6^ cells/mL in 96-well microtitre plates and incubated overnight at 37°C with 5% CO_2_. Cells were activated with 1 μg/ml of LPS (Sigma) and co-incubated with different concentrations (100, 50, 25 and 12.5 μg/ml) of the extracts or quercetin (positive control). Cells were then incubated for 24 h at 37°C with 5% CO_2_. Cells treated with LPS alone served as negative control.

#### Determination of Nitric Oxide Production

The activity of plant extracts against nitric oxide produced from the RAW 264.7 macrophages was done by measuring the amount of nitrite produced by the cells. The release of nitric oxide from the RAW 264.7 macrophages was determined by measuring the concentration of nitrite in culture supernatants by the Griess reaction assay as previously described (Mu et al., 2001).

#### Determination of Cell Viability

To ensure that the activity of the nitric oxide inhibition by extracts was not due to cytotoxicity, a cytotoxicity assay using 3-(4,5-dimethythiazol-2-yl)-2,5-diphenyl tetrazolium bromide (MTT) was performed (Mosmann, 1983) with slight modifications as described above. The percentage of cell viability was calculated by comparing the absorbance in the plant extract-treated wells to the negative control (cells treated with LPS only were recorded as being 100% viable).

### Immunomodulatory Assay

#### Preparation of Samples

A single concentration of the acetone plant extracts (50 μg/ml) was used in this assay to determine their immunomodulatory activities. This concentration was chosen because it was safe to the cells with the crude extract and fractions. The sample concentrations were prepared freshly on the day of experiment in cell culture medium.

#### Preparation of Cells

Human leukaemia monocytic THP-1 cells (ATCC, TIB-202) were maintained in RPMI-1640 medium supplemented with 10% FBS and 1% PSF in 75 cm^2^ culture flasks. The cell suspension supplemented with 0.10 μg/ml phorbol 12-myristate 13-acetate (PMA; Sigma) was seeded into 96-well tissue culture microtitre plates at a concentration of 2 × 10^5^ cells/mL and cultured for 72 h at 37°C in an incubator containing 5% CO_2_ to allow for attachment and differentiation of the monocytes into macrophages. The PMA-containing medium was discarded, and the differentiated THP-1 cells were washed with PBS. Lipopolysaccharide (100 μl of 1 μg/ml solution) prepared in the complete medium was added to the wells to stimulate cytokine production. After 60 min, 100 μl of the extracts (50 μg/ml) were added to the LPS-stimulated cells in duplicate. After 48 h incubation, the supernatant was collected and stored at −70°C.

#### THP-1 Cell Viability

The viability of the THP-1 cells was determined after treatment with the extracts using the MTT assay (Mosmann, 1983; McGaw et al., 2007) as described above. The percentage of cell growth inhibition was calculated with reference to untreated cells.

#### Cytokine Detection via Cytometric Bead Array Analysis

The influence of the extracts on the production of interleukin-1β (IL-1β), interleukin-6 (IL-6), interleukin-10 (IL-10), Tumor Necrosis Factor (TNF-α), and Interleukin-12p70 (IL-12p70) protein levels in the cell culture supernatants was determined using a commercial kit (CBA Human Inflammatory Cytokines Kit manufactured by BD Biosciences) following the manufacturer’s guidelines. Acquisition of data was done with a BD Accuri flow cytometer and the data was analysed with the FCAP Array software.

### Statistical Analysis

Statistical analysis was performed with GraphPad Prism (Version 5) using one-way analysis of variance (ANOVA). The Dunnett’s multiple comparison test was performed to identify significance compared to control values. *p* value less than 0.05 was considered significant. The data is expressed as the mean ± standard deviation.

## Results

### Antimycobacterial Assay and Cytotoxicity

The antimycobacterial activity of the extract and fractions, tested against four non-tuberculous mycobacteria (*M. smegmatis*, *M. fortuitum*, *M. aurum* and *M. bovis* BCG) and a pathogenic strain (*M. tuberculosis* H37Rv) is presented in Table 1. The MIC values of the acetone crude extract and fractions of *P. capensis* ranged from 39 to 1,250 μg/ml against all the tested organisms with the crude extract and hexane fraction having the best MIC value (39 μg/ml) against *M. aurum* and *M. smegmatis* respectively while the n-butanol fraction had the weakest MIC value of 1,250 μg/ml against *M. fortuitum* (Table 1). The cytotoxicity screening of the crude extract and fractions against Vero kidney cells (Table 1) revealed LC_50_ values ranging from 60 to 290 μg/ml. The crude extracts had relative higher toxicity against the tested cell line while the hexane fraction was the least cytotoxic at the highest concentration. The SI values ranged from 0.06 to 7.4 with the hexane fraction having the highest value of 7.4 against *M. smegmatis* (Table 1). Total antibacterial activity (TAA) indicates the volume to which the active constituents present in the extract or fraction can be diluted and still inhibit the growth of the tested organisms. The n-butanol fraction had the highest total activity of 24.9 ml/g against *M. fortuitum* while the chloroform fraction had the least total activity of 0.2 ml/g (Table 2). The mean TAA of the fractions ranged from 0.3 to 10.3 ml/g while the mean TAA for the crude extract of *P. capensis* was 7.1 ml/g.

**TABLE 1:** Minimum inhibitory concentration (MIC), cytotoxicity against Vero kidney cells (LC_50_), and selectivity index (SI) of acetone extract and fractions of *Psychotria capensis*.

Plant extracts	Microorganisms										
	LC_50_	*M. aurum*		*M. bovis* BCG		*M. smegmatis*		*M. fortuitum*		H37Rv	
		MIC	SI	MIC	SI	MIC	SI	MIC	SI	MIC	SI
PC	60	39	1.5	312	0.2	156	0.4	156	0.4	156	0.4
HF	290	312	0.93	312	0.9	39	7.4	312	0.9	78	3.7
CF	250	156	1.6	312	0.8	156	1.6	156	1.6	156	1.6
n-butF	70	625	0.11	312	0.2	78	0.9	1,250	0.06	312	0.2
Rif	na	0.01	na	0.12	na	0.72	na	0.01	na	0.03	na
Doxorubicin	0.2		na		na		na		na		na

All values are in µg/ml. PC, *Psychotria capensis* acetone extract; HF, hexane fraction; CF, chloroform fraction; n-butF, n-butanol fraction; Rif, Rifampicin.

**TABLE 2:** Total antibacterial activity in mL/g (TAA) of the acetone extract and fractions of *P. capensis* against *Mycobacterium* species.

Extract	*M. aurum*	*M. bovis* BCG	*M. smegmatis*	*M. fortuitum*	H37Rv
HF	7.9	7.9	1.0	7.9	2.0
CF	0.2	0.5	0.2	0.2	0.2
n-butF	12.4	6.2	1.6	24.9	6.2
PC	1.7	13.6	6.8	6.8	6.8

PC, *Psychotria capensis* acetone extract; HF, hexane fraction; CF, chloroform fraction, n-butF, n-butanol fraction.

### Anthelmintic Assay

The percentage egg hatch inhibition of the extract, fractions as well as the drug control had a concentration dependent effect. At the highest tested concentration (2,500 μg/ml), the chloroform and butanol fractions inhibited egg hatching by 100%. The EC_50_ values ranged from 160 to 630 μg/ml with the hexane fraction having the best EC_50_ value followed by *P. capensis* crude extract (Table 3). Albendazole had EC_50_ of 0.02 μg/ml. Of all the extracts, the hexane fraction had the best selectivity index of 1.81.

**TABLE 3:** Lethal concentrations required to inhibit 50% of *Haemonchus contortus* egg hatching of acetone extract and fractions of *P. capensis* and their selectivity indices (LC_50_/EC_50_).

Extract	EC_50_ (µg/ml)	Selectivity index (SI)
PC	230	0.26
HF	160	**1.81**
CF	630	0.63
n-butF	630	0.11
Albendazole	0.02	ND

PC, *Psychotria capensis* acetone extract; HF, hexane fraction; CF, chloroform fraction; n-butF, n-butanol fraction.

Values in bold indicate promising activity.

### GC-MS Analysis of the Crude Extract and Hexane Fraction of *Psychotria capensis*

The phytoconstituents present in the acetone extract of *P. capensis* and hexane fraction were detected by GC-MS analysis and identified by mass spectrometry. The GC-MS analysis revealed 12 compounds for both the acetone crude extract of *P. capensis* and the hexane fraction tentatively identified and confirmed with the library match with similarity values of 70% and above based on the peak area, retention time and molecular formula (Tables 4, 5). This is the first report on the chemical components and biological investigations of the hexane-soluble fraction of *P. capensis* to the best of our knowledge.

**TABLE 4:** Compounds detected in the acetone crude extract of *Psychotria capensis*.

S/N	Compound	RT (min)	Molecular formula	Similarity score (%)	MW (g/mol)	Chemical class
1	Methyl methacrylate	4.69	C_5_H_8_O_2_	86.7	100.12	Ester
2	Toluene	5.42	C_7_ H_8_	98.79	92.14	Aromatic hydrocarbon/arene
3	Furan, 2-methoxy-	7.61	C_5_ H_6_O_2_	80.64	98.10	Furan ether
4	1-Butanol	8.05	C_4_ H_10_O	89.25	74.12	Primary alcohol
5	Butanedioic acid, phenyl-	10.27	C_10_ H_10_ O_4_	80.78	194.18	Organic acids
6	2-Pentanone, 4- hydroxy-4-methyl-	12.35	C_6_H_12_O_2_	87.49	116.16	Acetone alcohol
7	Acetic acid	13.89	C_2_H_4_O_2_	84.61	60.01	Organic acid
8	Ethanone, 2- (formyloxy)-1-phenyl-	15.06	C_9_ H_8_O_3_	81.77	136.15	Phenyl ketone
9	Benzyl alcohol	20.24	C_7_H_8_O	79.49	108.14	Phenyl alcohol
10	Neophytadiene	20.85	C_20_H_38_	88.37	278.51	Diterpene
11	Bicyclo [2.2.1]heptane,1,3,3-trimethyl-	21.59	C_10_H_18_	74.39	138.25	Alkyne
12	Citronellyl isobutyrate	29.21	C_14_H_26_O_2_	80.45	212.33	Ester

**TABLE 5:** Compounds detected in the hexane fraction of *Psychotria capensis*.

S/N	Compound	RT (min)	Molecular formula	Similarity score (%)	MW (g/mol)	Chemical class
1	Methyl methacrylate	4.69	C_5_H_8_O_2_	86.70	100.12	Ester
2	Toluene	5.42	C_7_ H_8_	98.79	92.14	Aromatic hydrocarbon/Arene
3	3,4-Hexanedione, 2,2,5-trimethyl-	10.13	C_9_H_16_O_2_	73.66	156.22	Ester
4	Styrene	10.75	C_8_H_8_	86.55	104.15	Alkene
5	2-Pentanone, 4- hydroxy-4 methyl-	12.35	C_6_H_12_O_2_	76.89	116.16	Hydroxyl ketone
6	Benzene, 1,3-bis(1,1- dimethylethyl)-	13.45	C14H22	73.19	220.35	Arene
7	Ethanone, 2- (formyloxy)-1-phenyl-	15.06	C_9_H_8_O_3_	76.89	136.15	Phenyl ketone
8	2,3-Butanedione	15.47	C_4_H_6_O_2_	75.22	86.09	Vicinal diketone
9	1-Hexene, 3,5,5- trimethyl-	20.13	C_9_H_18_	79.49	126.24	Alkene
10	Benzyl alcohol	20.24	C_7_H_8_O	89.93	108.14	Aromatic alcohol
11	Neophytadiene	20.85	C_20_H_38_	98.02	278.51	Diterpene
12	3,7,11,15-Tetramethyl-2-hexadecen-1-ol	21.59	C_20_H_40_O	89.56	296.53	Acyclic diterpene

### Immune Modulatory Activity

Activation of macrophages with stimulants such as LPS can enhance the production of nitric oxide. RAW 264.7 macrophages were stimulated with LPS and treated with different concentrations of acetone extract and fractions of *P. capensis*. The NO assay results revealed that the acetone crude extracts and the fractions of *P. capensis* had a concentration dependent inhibition on NO production at concentrations of 12.5, 25, 50 and 100 μg/ml while the IC_50_ values ranged from 29.57 to 57.80 μg/ml (Table 6). The chloroform fraction had the highest percentage NO inhibition of 60% at the highest tested concentration (100 μg/ml) but a 34.47% cell viability against the macrophage cell line (Table 6). The hexane fraction had 54% NO inhibition with 96.53% cell viability at the highest tested concentration (Table 6). The hexane fraction also had the best IC_50_ value of 29.57 μg/ml (Table 6). The NO inhibitory activity of the hexane fraction was therefore not due to a general metabolic toxicity.

**TABLE 6:** Percentage cell viability and nitric oxide inhibitory concentration (IC_50_) of acetone extract and fractions of *Psychotria capensis*.

Samples	Concentration (µg/ml)	% Macrophage cell viability	IC_50_ (µg/ml)
PC	100	30.47	57.80
	50	63.93	
	25	79.85	
	12.5	87.13	
HF	100	96.53	**29.57**
	50	96.40	
	25	95.63	
	12.5	88.02	
CF	100	34.47	45.60
	50	59.85	
	25	76.45	
	12.5	86.45	
n-butF	100	85.23	31.37
	50	94.34	
	25	94.39	
	12.5	97.23	
Quercetin	100	64.41	7.92
	50	78.48	
	25	88.10	
	12.5	83.99	

All values are in µg/ml. PC, *Psychotria capensis* acetone extract; HF, hexane fraction; CF, chloroform fraction; n-butF, n-butanol fraction.

Values in bold indicate promising activity.

**FIGURE 1:**
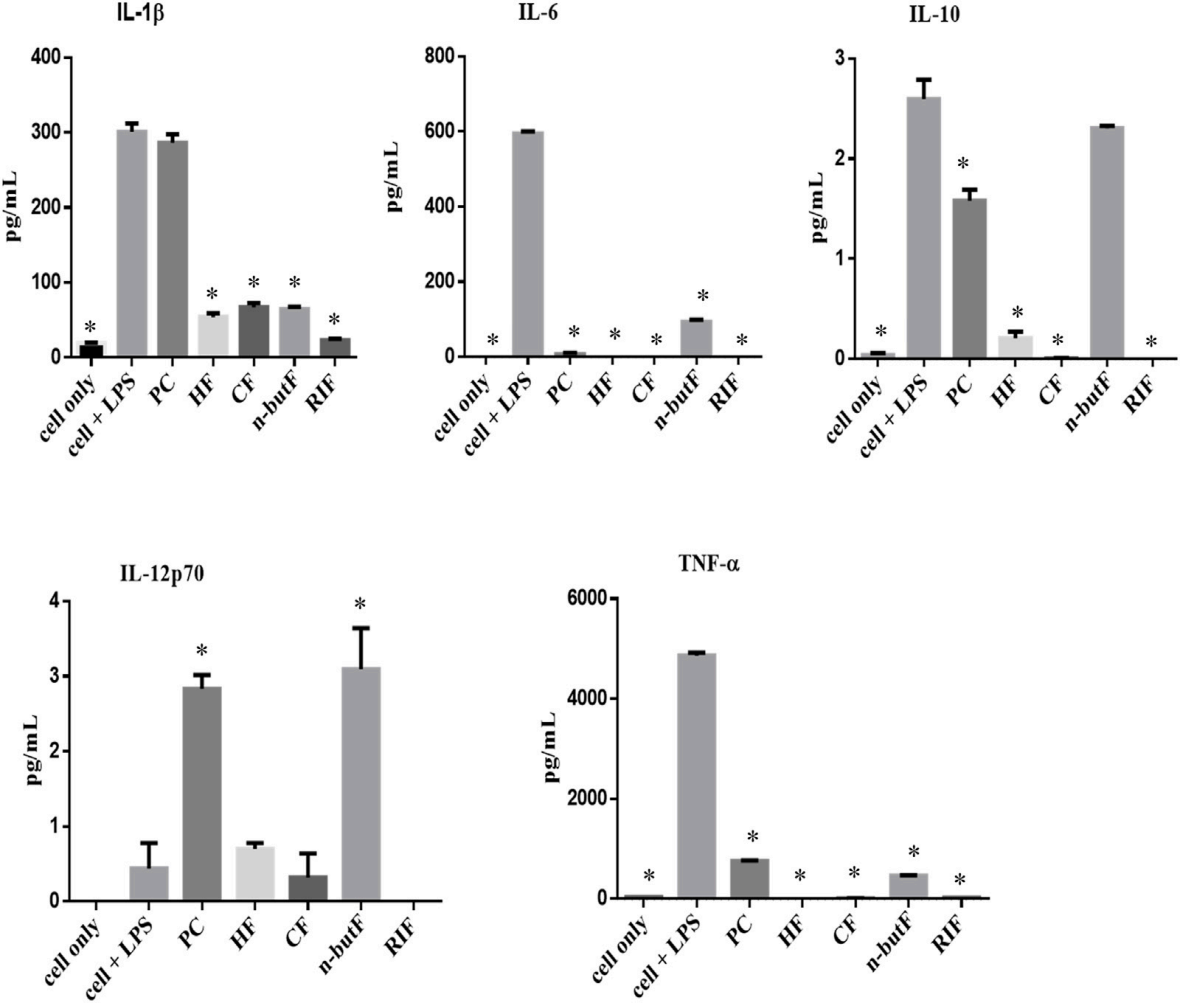
Cytokines produced by THP-1 cells treated with crude extract and fractions of cells only (cell + LPS) PC, P capensis acetone crude extract; HF, hexane fraction; CF, chloroform fraction; n-butF, n-butanol fraction; RIF, rifampicin. *denotes statistically signification difference between cells + LPS and the samples or cells only.

A particle-based immunoassay combined with flow cytometry was used to measure the levels of five cytokines, IL-1β, IL-6, IL-10, TNF-α and IL-12p70, in LPS stimulated monocytic THP-1 cells. The differentiated macrophages were treated with 50 μg/ml of acetone crude extract and fractions of *P. capensis* or rifampicin. Results obtained from the MTT assay revealed that none of the tested samples were cytotoxic to the THP-1 cell line with the LC_50_ values ranging from 40 to 450 μg/ml with the n-butanol fraction being the least cytotoxic. The supernatants were collected post 48 h treatment and the levels of cytokines in the cell culture media were quantified. All the fractions of *P. capensis* (HF, CF and n-butF) significantly (*p* < 0.05) decreased the expression of IL-1β compared to LPS stimulated cell only while the crude extract enhanced its expression but not significantly (Figure 1). All the tested samples including rifampicin significantly inhibited the expression of IL-6 and TNF-α. The crude extract of *P. capensis* and n-butanol fraction significantly (*p* < 0.05) promoted IL-12p70 production while rifampicin completely suppressed the expression of this pro-inflammatory cytokine (Figure 1). All the tested samples except the n-butanol fraction significantly reduced the expression of IL-10 compared to cells + LPS (Figure 1).

## Discussion

Tuberculosis remains a significant cause of morbidity and mortality in many developing countries globally. Helminth infections are endemic in developed and underdeveloped countries and are known to cause chronic and long-standing infection with the ability to impair both innate and adaptive immune response to pathogens such as *M. tuberculosis* (Babu and Nutman, 2016). The impact of helminth infection on the progression of active tuberculosis or anti-TB treatment and vaccination has been reported (Elias et al., 2006; George et al., 2015; Babu and Nutman, 2016). It is pertinent to consider the effect of coincidence with helminth infection during vaccine studies or development of anti-TB therapy, most importantly in helminth-endemic regions of the world (Babu and Nutman, 2016).

Following our previous findings on the antimycobacterial, antioxidant and immunomodulatory activity, as well as synergistic effects in combination with rifampicin, and apoptosis inducing activities of some Rubiaceae species, *Psychotria capensis* had excellent pharmacological activity (Aro et al., 2015; Aro et al., 2016; Aro et al., 2019). Therefore, in this study, the antimycobacterial, anthelmintic, anti-inflammatory, and immune-modulatory activities from the fractions and crude extract of *P. capensis* were determined in more depth in order to identify the fractions that might contain the active compounds. Crude extracts and fractions with an MIC <100 μg/ml are considered as having good activity with promising potential for further studies (Eloff, 1998a). If the MIC value is >100–625 μg/ml, it is considered moderate, and weak if MIC is >625 μg/ml (Sánchez and Kouznetsov, 2010). Based on the antimycobacterial assay, the hexane fraction, and the acetone extract of *P. capensis* had the best MIC value of 39 μg/ml against *M. smegmatis* and *M. aurum* respectively. All the tested fractions and the crude extract of *P. capensis* had good to moderate antimycobacterial activities against both non-pathogenic (*M. aurum*, *M. bovis* BCG, *M. smegmatis* and *M. fortuitum*) and pathogenic *M. tuberculosis* H37Rv strains except the n-butanol fraction which had weak antimycobacterial activity against *M. fortuitum* at the highest concentration tested. The crude acetone extract of *P. capensis* had moderate relative cytotoxicity against Vero kidney cells with an LC_50_ value of 60 μg/ml. Interestingly, fractionation of the crude extract reduced the relative cytotoxicity as observed with the chloroform and hexane fractions with LC_50_ values of 290 and 250 μg/ml respectively. While toxicity was reduced, there was a consequent increased antimycobacterial activity against *M. smegmatis* by both the hexane and butanol fractions, while there was better activity against the virulent H37Rv strain by the hexane fraction compared to the acetone crude extract thereby leading to a higher selectivity index. The highest SI value of 7.4 was obtained with the hexane fraction against *M. smegmatis.* While this indicates that the fraction was more active against the mycobacteria than toxic to the mammalian cells, SI values greater than or equal to 10 are preferable to indicate that there is a possible therapeutic use (Vonthron-Sénécheau et al., 2003).

Plants from the Psychotrieae tribe were shown to be major producers of alkaloids and have been considered as a ‘hot genus’ owning to their reported pharmacological activities (Moraes et al., 2011). The relative cytotoxicity recorded in the crude extract of *P. capensis* could be due to the presence of alkaloids peculiar to the *Psychotria* genus. Further investigations relating to future therapeutic use of the *P. capensis* extract or fractions need to include more comprehensive toxicity studies, both *in vitro* and *in vivo* owing to the known occurrence of potentially toxic alkaloids in the genus. Total antibacterial activity (TAA) reflects to what volume the mass extracted from 1 g of an extract or fraction can be diluted and still have inhibitory antimicrobial activity (Eloff, 2004). TAA can also indicate if activity was lost or gained in biological activities at each step of fractionation. The n-butanol and hexane fractions had a better TAA for *M. aurum* and *M. fortuitum*, showing that sometimes, fractionation of an extract may enhance antimicrobial potency. In some cases, however, fractionation of the crude extract of a Rubiaceae species could lead to loss of activity (Aro et al., 2019). The loss or gain may be due to processes such as photo-oxidation or synergistic interaction respectively between the plant fractions or compounds (Eloff, 2004).

Studies conducted in experimental models and human infections show that worm-infected subjects have a high risk of contracting tuberculosis and failure of therapy or vaccination against TB during co- or pre-infection with helminths has an immunological basis (Potian et al., 2011; Monin et al., 2015). The *in vitro* anthelmintic potential of the crude extract and fractions of *P. capensis* was evaluated in this study. Plant extracts having an EC_50_ above 6 mg/ml can be considered to possess weak anthelmintic activity due to the fact that it is extremely difficult to achieve such a high concentration *in vivo* (Adamu et al., 2013). Based on the foregoing, all the tested samples had excellent activity (EC_50_ values ranging from 230 to 630 μg/ml) against *H. contortus* in this study. Diterpenes and esters were isolated from the acetone leaf extracts of *P*. *capensis* while diterpene and acyclic diterpenes were isolated from the hexane fractions of *P. capensis*. Compounds such as β-sitosterol and the carotenoid derivative lutein have been previously reported to be isolated from *P. capensis* (Carvalho et al., 2013). Compounds including β-sitosterol, carotenoid derivatives and alkaloids have been reported to be isolated from *Psychotria* species (Marques de Oliveira et al., 2013) and these are known to have anthelmintic activity (Wang et al., 2010; Ali et al., 2011). Saponins possess the ability to disrupt cell membranes, thereby increasing cell permeability by interacting with membrane-associated sterols (Price et al., 1987). The results obtained from this study are comparable to the values reported by Adamu and others where the acetone crude extract of *Heteromorpha trifoliata* and *Maesa lanceolata* had a 100% egg hatch inhibition effect on *H. contortus* and EC_50_ values less than 1,000 μg/ml (Adamu et al., 2013). The observed anthelmintic activity of the acetone crude extracts of *Heteromorpha trifoliata* and *Maesa lanceolata* was attributed to the presence of saponins and alkaloids reported to be present in the plants (Lukhoba et al., 2006; Tadesse et al., 2009). The *in vitro* anthelmintic activities of the acetone crude extract and fractions of *P. capensis* have not been previously reported. These results support the traditional use of the plant to treat gastric complaints, although further studies are required on different plant parts, and in animal models, to confirm anthelmintic efficacy.

GC-MS analysis of the acetone crude extract and hexane fraction of *P. capensis* leaves tentatively identified several components which may be responsible for the bioactivities reported in this study. It should be kept in mind that using similarity scores does not rule out false compound identification (Kim and Zhang, 2015). Future research on the chemical composition of *P. capensis* extracts should focus on calculating the Kovats index values to verify that the value is consistent with the compound identified by the NIST database. Compounds such as neophytadiene and citronellyl isobutyrate tentatively identified in the extract and fraction in this study most likely act in a synergistic way to confer the activity reported in this study. Neophytadiene and other compounds isolated from the hexane fraction of *Bursera simarubab* leaves were reported to be potentially responsible for the anti-inflammatory effects of plant (Carretero et al., 2008). In another study, the anti-inflammatory activity noted in the crude ethanol extract of *Chrysopogon aciculatus* was attributed to citronellyl isobutyrate, a constituent compound identified in the plant (Zihad et al., 2018). Neophytadiene has been recorded to have antimicrobial as well as anti-inflammatory activity (ChEBI, 2021). Further chemical and bioactivity studies should be undertaken to conclusively identify the bioactive compounds in *P. capensis*.

The production of nitric oxide in combination with reactive nitrogen and oxygen is involved in many biological processes with the ability to enhance bactericidal activities in activated macrophages. However, excessive production of reactive oxygen and nitrogen intermediates could lead to inflammation (Bogdan et al., 2000). Therefore, inhibition of NO production is desired to limit damaging effects of inflammation in the host. The selected samples screened in this study had a concentration-dependent NO inhibitory effect with the hexane fraction having the highest percentage NO inhibition (93%) and best IC_50_ value of 29.57 μg/ml (Table 6). In response to pro-inflammatory agents such as LPS, inducible nitric oxide synthase (iNOS) can act as a precursor for NO production in order to promote inflammation (Lu et al., 2008). Natural compounds present in medicinal plants have been reported to be potent inhibitors of iNOS expression in LPS-activated macrophages (Son et al., 2000; Razali et al., 2014). The U.S. National Cancer Institute plant screening program indicates that crude plant extracts having IC_50_ value less than 20 μg/ml upon incubation for 48–72 h are considered to be cytotoxic (Lee and Houghton, 2005). Based on this criterion, the crude extract and fractions of *P. capensis* can be said to be relatively non-cytotoxic at the tested concentrations against Vero kidney, RAW 264.7 mouse macrophages and THP-1 human cell lines. Significantly, fractionation was able to reduce the cytotoxic effects on the tested cell lines as observed with higher LC_50_ values ranging from 70 to 690 μg/ml against the Vero kidney cells and THP-1 cells respectively.

Most helminth parasites are deleterious to the health of their hosts, especially when there is co-infection with mycobacteria which promotes immune polarisation that can lead to progression of mycobacterial infections (Lucena et al., 2017). A hallmark of helminth infections, both in experimental models and human infection is the generation of profound T helper (Th) 2 and T regulatory cell responses that enhance the pathogenesis of *M. tuberculosis* infections (Anthony et al., 2007; Potian et al., 2011). Monocytic cells such as THP-1 cells can either be physiologically stimulated (with IFN-γ or LPS) or through exogenous chemical phorbol esters such as phorbol-12-myristate-13-acetate (PMA) to undergo macrophage polarization. Activation of macrophages by LPS enhances the production of proinflammatory mediators and cytokines such as TNF-α and the IL family (Dhar et al., 2018). The amount of IL-1β, IL-6, IL-10, TNF-α and IL-12p70 secreted into the cell culture media was quantified using a flow cytometer post 48 h of treatment of LPS stimulated THP-1 cells with the crude extract and fractions of *P. capensis*. The assayed cytokines are classified as pro-inflammatory cytokines (IL-1β, IL-6, TNF-α and IL-12p70) produced by the Th-1 cells and IL-10, an anti-inflammatory cytokine often produced by Th-2 cells. The production of the IL-12p70 cytokine has been reported for its protective role against various intracellular pathogens including those caused by mycobacteria (Wallington et al., 2018). Of interest in this study is the stimulatory effect of n-butanol fractions and crude extract of *P. capensis* on IL-12p70 which was better than that of the conventional anti-TB drug, rifampicin. The immunological hallmark of helminth infections is their ability to induce Th-2 associated immune responses characterized by the presence of the IL-4, IL-5, IL-9, IL-10, and IL-13 (Babu and Nutman, 2016). The inhibitory effect of all the tested samples on IL-10 is noteworthy, with the hexane fraction having the best inhibitory effect when compared with the untreated LPS-stimulated macrophages. However, the selected samples significantly decreased the expression of IL-1β, IL-6 and TNF-α. Pro-inflammatory cytokines such as IL-1β, IL-6 and IL-23 can be produced when macrophages are activated by TNF-α (Mosser and Edwards, 2008). TNF-α plays a critical role in the control of mycobacteria, but excessive production of this important cytokine could result in progression of disease and severe tissue damage (Mootoo et al., 2009). Similarly, although NO may be anti-inflammatory under normal conditions, it is considered to be a pro-inflammatory mediator inducing inflammation following over-production in abnormal circumstances (Sharma et al., 2007). Inhibition of this response by the *P. capensis* extract and fractions as discussed earlier is a useful characteristic. IL-6 is a cytokine with many roles important for both innate and adaptive immune responses and also plays a regulatory role in immune responses, hence acting as a pro- or an anti-inflammatory cytokine. Its regulatory role involves the reduction of TNF-α production (Wong and Ustunol, 2006). The reported inhibitory effect of all the fractions and crude extract of *P. capensis* on IL-6 and TNF-α could be beneficial as excessive production of both IL-6 and TNF-α could be detrimental to the host. The results obtained from this study are comparable to the data reported by Aro and others where the acetone crude extracts of some species belonging to the Rubiaceae family inhibited the expression of TNF-α and IL-10. This therefore indicates a selective Th-1 response (Aro et al., 2019).

The ability of the fractions and crude extract of *P. capensis* to induce the production of Th-1 cytokines and inhibit expression of Th-2 cytokines can possibly aid the immune response in combating and inhibiting the proliferation of mycobacteria coupled with the anthelmintic activities. To the best of our knowledge, this is the first report of the antimycobacterial and anthelmintic activities of the fractions and acetone extract of *P. capensis*. Helminth infections in humans have been proven to suppress NO synthase by inhibiting activation of macrophages, downregulating T cells and inhibiting apoptosis of dendritic and T cells (Babu and Nutman, 2016). A previous study showed that the acetone crude extract of *P. capensis* had a remarkable inhibitory effect on 15-lipoxygenase, coupled with promising free radical scavenging ability and good apoptotic induction on THP-1 cells (Aro et al., 2019).

These reported biological activities could possibly be the mode of action for *P. capensis* to inhibit helminth and mycobacterial infections and boost the host immune response to indirectly combat inflammatory infections such as helminthiasis and TB. While studies of helminth co-infection with TB and their deleterious effects are lacking in South Africa, elsewhere on the African continent there is accumulating evidence that prevention of helminthiasis might be part of the solution to the pandemics of HIV/AIDS and TB (Borkow and Bentwich, 2004). Impeding development of drug resistance may be promoted by developing complementary herbal based medications to reduce helminth infestation and re-infestation, in turn reducing the burden of TB and subsequent development of resistance.

## Conclusion

Findings from this study reveal that the hexane fraction of *P. capensis* had the best dual activity against *Mycobacterium* spp. and the helminth parasite *Haemonchus contortus*, as well as a profound Th-2 inhibitory effect *in vitro*. The good nitric oxide inhibitory effect of the crude extract of *P. capensis* and hexane fraction support their therapeutic potential in inflammatory disease. More studies are needed to evaluate the ability of the fraction and crude extract of *P. capensis* to inhibit the growth of mycobacteria and helminths in a co-infection intracellular model. Likewise, conducting an *in vivo* study will be valuable.

In conclusion, the hexane fraction of *P. capensis* has the potential to act as an adjuvant in the treatment of inflammatory diseases. The bioactive principles present in the hexane fraction could contribute towards standardization of herbal formulations to combat helminth-TB co-morbidity. Isolation and identification of the bioactive compounds responsible for the observed pharmacological activities in the hexane fraction is ongoing.

## Data Availability

The raw data supporting the conclusions of this article will be made available by the authors, without undue reservation.
